# Elevated mRNA-levels of distinct mitochondrial and plasma membrane Ca^2+^ transporters in individual hypoglossal motor neurons of endstage SOD1 transgenic mice

**DOI:** 10.3389/fncel.2014.00353

**Published:** 2014-11-14

**Authors:** Tobias Mühling, Johanna Duda, Jochen H. Weishaupt, Albert C. Ludolph, Birgit Liss

**Affiliations:** ^1^Department of Applied Physiology, Institute of Applied Physiology, Ulm UniversityUlm, Germany; ^2^Department of Neurology, Ulm UniversityUlm, Germany

**Keywords:** mCU, choline acetyltransferase ChAT, UCP2, Letm1, NCX1, mitochondrial DNA, ND1, GFAP

## Abstract

Disturbances in Ca^2+^ homeostasis and mitochondrial dysfunction have emerged as major pathogenic features in familial and sporadic forms of Amyotrophic Lateral Sclerosis (ALS), a fatal degenerative motor neuron disease. However, the distinct molecular ALS-pathology remains unclear. Recently, an activity-dependent Ca^2+^ homeostasis deficit, selectively in highly vulnerable cholinergic motor neurons in the hypoglossal nucleus (hMNs) from a common ALS mouse model, the endstage superoxide dismutase SOD1^G93A^ transgenic mouse, was described. This functional deficit was defined by a reduced hMN mitochondrial Ca^2+^ uptake capacity and elevated Ca^2+^ extrusion across the plasma membrane. To address the underlying molecular mechanisms, here we quantified mRNA-levels of respective potential mitochondrial and plasma membrane Ca^2+^ transporters in individual, choline-acetyltransferase (ChAT) positive hMNs from wildtype (WT) and endstage SOD1^G93A^ mice, by combining UV laser microdissection with RT-qPCR techniques, and specific data normalization. As ChAT cDNA levels as well as cDNA and genomic DNA levels of the mitochondrially encoded NADH dehydrogenase ND1 were not different between hMNs from WT and endstage SOD1^G93A^ mice, these genes were used to normalize hMN-specific mRNA-levels of plasma membrane and mitochondrial Ca^2+^ transporters, respectively. We detected about 2-fold higher levels of the mitochondrial Ca^2+^ transporters MCU/MICU1, Letm1, and UCP2 in remaining hMNs from endstage SOD1^G93A^ mice. These higher expression-levels of mitochondrial Ca^2+^ transporters in individual hMNs were not associated with a respective increase in number of mitochondrial genomes, as evident from hMN specific ND1 DNA quantification. Normalized mRNA-levels for the plasma membrane Na^+^/Ca^2+^ exchanger NCX1 were also about 2-fold higher in hMNs from SOD1^G93A^ mice. Thus, pharmacological stimulation of Ca^2+^ transporters in highly vulnerable hMNs might offer a neuroprotective strategy for ALS.

## Introduction

Amyotrophic Lateral Sclerosis (ALS) is the most common motor neuron (MN) degenerative disease, with an adult onset and an annual incidence of 1–3 cases per 100,000 people worldwide (Ludolph et al., 2012; Valori et al., 2014). Typical histopathological hallmark of ALS is the loss of upper and lower motor neurons, which is accompanied by reactive gliosis (Valori et al., 2014). Although first described in 1869 (Charcot and Joffroy, 1869), the causes of ALS remain largely unknown and, as effective long-term treatment strategies are not available, most patients die 3–5 years after symptom-onset (Hardiman et al., 2011; Vucic et al., 2014). There is a family history in around 10% of ALS patients, that can be attributed to several gene defects (Renton et al., 2013). Most common is an abnormal hexanucleotide expansion of the chromosome 9 open reading frame 72 gene (C9ORF72), present in many familial as well as some sporadic ALS cases (DeJesus-Hernandez et al., 2011; Renton et al., 2011; Haeusler et al., 2014). However, causes for neurodegeneration in most sporadic ALS patients still remain mostly unresolved (Vucic et al., 2014). Disease mechanisms of both, sporadic and familial ALS, share common pathogenetic features, in particular glutamate excitotoxicity, calcium (Ca^2+^) overload, mitochondrial dysfunction, oxidative stress and protein dysfunction and/or aggregation, e.g., particularly of the 43-kDa trans-activating response region binding protein (TDP-43) or cytoplasmic Cu^2+/^Zn^2+^-superoxide dismutase 1 (SOD1) (Neumann et al., 2006; Ilieva et al., 2009; Ferraiuolo et al., 2011; Lee et al., 2012; Matus et al., 2013; Rotunno and Bosco, 2013; Muyderman and Chen, 2014; Tadic et al., 2014). There is also strong evidence for a crucial role of astroglia cells for MN degeneration in ALS (Valori et al., 2014). In both, ALS patients and its transgenic animal models, there is evidence of ubiquitinated protein inclusions in MNs as well as in glial cells (Bruijn et al., 1997; Pasinelli et al., 2000; Mendonça et al., 2006).

Independent of the cause of ALS, one neuro-pathological hallmark of the disease is the differential vulnerability of MN populations to neurodegenerative triggers, e.g., MNs in spinal cord or hypoglossal nucleus are particularly vulnerable to ALS-trigger factors, while other MN populations, e.g., in particular in the oculomotor nucleus, remain relatively spared (Cleveland and Rothstein, 2001; Kanning et al., 2010; Kaplan et al., 2014). Vulnerable MNs display low endogenous Ca^2+^ buffering capacity due to a lack of cytosolic Ca^2+^ binding proteins (like calbindin_d28k_ or parvalbumin) (von Lewinski and Keller, 2005), accompanied by expression of AMPA glutamate receptor subtypes that are highly permeable to Ca^2+^ (Van Den Bosch et al., 2000; Grosskreutz et al., 2010). Thus, these vulnerable MNs depend particularly on mitochondrial Ca^2+^ uptake to recover from transient Ca^2+^ increase during electrical activity (Grosskreutz et al., 2007; Jaiswal and Keller, 2009). However, mitochondria in highly vulnerable MNs show substantial functional and morphological changes in ALS animal models and human patients (Kawamata and Manfredi, 2010; Barrett et al., 2011; Martin, 2011; Cozzolino and Carrì, 2012; Vehviläinen et al., 2014). In particular, increased cytosolic Ca^2+^ transients and significantly reduced mitochondrial Ca^2+^ uptake have been described in ALS mouse models (Jaiswal and Keller, 2009; Coussee et al., 2011). The mitochondrial membrane potential, the driving force for mitochondrial Ca^2+^ uptake, has been described to be depolarized, and/or Ca^2+^ induced depolarization was increased (Carrì et al., 1997; Damiano et al., 2006; Jaiswal and Keller, 2009; Nguyen et al., 2009). By functional comparison of MNs from the hypoglossal (hMN) and from the oculomotor nucleus (oMN) in the most commonly utilized mouse model of ALS that express a human disease causing G93A SOD1 mutation (SOD1^G93A^ mice), we recently identified a Ca^2+^ homeostasis deficit, selectively in highly vulnerable hMNs at disease endstage (Fuchs et al., 2013). More precisely, in response to elevated electrical activity, a reduced mitochondrial Ca^2+^ uptake, and an enhanced Ca^2+^ extrusion across the plasma membrane was observed in hMNs but not oMNs (Fuchs et al., 2013).

The main assumed uptake route of Ca^2+^ into mitochondria is the mitochondrial Ca^2+^ uniporter (mCU), driven by the mitochondrial membrane potential (Drago et al., 2011). It consists of a pore-forming subunit, named mitochondrial Ca^2+^ uniporter (MCU) and at least two regulatory subunits, mitochondrial Ca^2+^ uptake 1 (MICU1) and mitochondrial Ca^2+^ uniporter regulator 1 (MCUR1) (Perocchi et al., 2010; Baughman et al., 2011; de Stefani et al., 2011; Mallilankaraman et al., 2012a; Marchi and Pinton, 2014). mCU has a relatively low Ca^2+^ sensitivity, and probably achieves mitochondrial Ca^2+^ import mainly at endoplasmic reticulum (ER) mitochondria microdomains, where Ca^2+^ concentrations are high enough (Drago et al., 2011). The mitochondrial uncoupling proteins UCP2 and UCP3 can also contribute to the mitochondrial Ca^2+^ uptake machinery. First reported to be a component of the uniporter itself (Trenker et al., 2007), they were later supposed to operate independently of other Ca^2+^ uptake pathways, particularly when Ca^2+^ is released from the ER (Waldeck-Weiermair et al., 2011). In addition, the high Ca^2+^ affine leucine zipper EF-hand containing transmembrane protein 1 (Letm1) is supposed to function as mitochondrial Ca^2+^/H^+^ exchanger at the relatively low cytosolic concentration increases across the plasma membrane that follow Ca^2+^ depletion of the ER (store operated calcium entry, SOCE) (Jiang et al., 2009; Waldeck-Weiermair et al., 2011; Nowikovsky et al., 2012). The mitochondrial Na^+^/Ca^2+^exchanger (MNCX) achieves Ca^2+^ efflux from mitochondria under physiological conditions (Palty et al., 2010; de Marchi et al., 2014) but might revert its operation mode when mitochondria undergo pathological de- or hyperpolarization (Kim and Matsuoka, 2008; Chinopoulos and Adam-Vizi, 2010). Apart from mitochondrial Ca^2+^ uptake, the sarco-endoplasmic reticulum Ca^2+^ ATPases (SERCA) are transporting Ca^2+^ into the ER, and thus provide additional Ca^2+^ clearance capacity (Hovnanian, 2007; Chaudhari et al., 2014; Hajnóczky et al., 2014). Furthermore, Ca^2+^ clearance via the plasma membrane is present and mediated by the plasma membrane Ca^2+^ ATPases (PMCA1-4) (Strehler, 2013) and the Na^+^/Ca^2+^ exchangers (NCX1-3) (Sharma and O'Halloran, 2014). While NCX1-3 mediate extrusion of steep Ca^2+^ increases after cell stimulation via the plasma membrane, PMCA isoforms are regarded as “fine tuners” of cytosolic Ca^2+^ extrusion (Brini and Carafoli, 2011).

To molecularly define the described complex altered functional Ca^2+^ clearance phenotype in hMNs of endstage SOD1^G93A^ mice (Fuchs et al., 2013), here we examined mRNA-levels of all described potential mitochondrial (MCU/MICU1/MCUR1, Letm1, UCP2/3, MNCX) and plasma-membrane (PMCA1-4, NCX1-3) Ca^2+^ transport proteins in choline-acetyltransferase (ChAT) positive hMNs of endstage SOD1^G93A^ and wildtype (WT) mice, by combining UV laser microdissection (UV-LMD) with quantitative RT-PCR analysis. To assess if a possible cell-specific transcriptional Ca^2+^ transporter dysregulation is associated with an altered number of mitochondria/mitochondrial genomes in SOD1^G93A^ mice (Keeney and Bennett, 2010), we quantified mitochondrial genomic DNA in individual hMNs via quantification of the mitochondrially encoded NADH dehydrogenase subunit 1 (ND1) gene (He et al., 2002; Bender et al., 2006; Krishnan et al., 2007). As cDNA levels for ChAT as well as cDNA and genomic DNA levels for ND1 were not altered in hMNs from SOD1^G93A^ mice compared to WT, we utilized these genes for normalization of respective qPCR expression-data for plasma membrane and mitochondrial Ca^2+^ transporters. With this stratified analysis, we detected a selective transcriptional up-regulation of the mitochondrial MCU/MICU1 complex, similar as previously described (Fuchs et al., 2013), as well as of Letm1, UCP2, and the plasma membrane transporter NCX1. These findings point to an activity-dependent increased need of Ca^2+^ clearance capacity in hMNs of endstage SOD1^G93A^ mice that is only partly met by an increased expression of mitochondrial Ca^2+^ transporters. Accordingly, Ca^2+^ extrusion via the plasma-membrane is elevated in hMNs of endstage SOD1^G93A^ mice—not only functionally (Fuchs et al., 2013), but also at the molecular level, via elevated NCX1 expression. Thus, pharmacological stimulation of Ca^2+^ transporters might offer a novel neuroprotective strategy for highly vulnerable MNs in ALS.

## Materials and methods

### Ethical approval

All animal procedures were approved by Regierungspräsidium Tübingen, Germany (AZ 35/9185.81-3 TV No. 1090, and O-147), and conducted according to the guidelines of the German Tierschutzgesetz.

### Mice

For all experiments, male transgenic mice of the strain B6SJL-TgN(SOD1-G93A) (Jackson Laboratory, Bar Harbor, US) and wildtype (WT) littermates of the same genetic background were used (Gurney et al., 1994). Mice were bred in Ulm in respective in-house breeding facility and genotyped according to the protocol recommended by Jackson Laboratory. For analyses, SOD1^G93A^ mice between P115 and P145 were used after they were no longer able to pass a paw grip endurance test (clinical score 4, referred to as endstage) (Solomon et al., 2011). Data were derived from six individual SOD1^G93A^ and six respective age-matched WT mice.

### Tissue preparation, UV laser microdissection (UV-LMD) and reverse transcription (RT)

**Figure 1:**
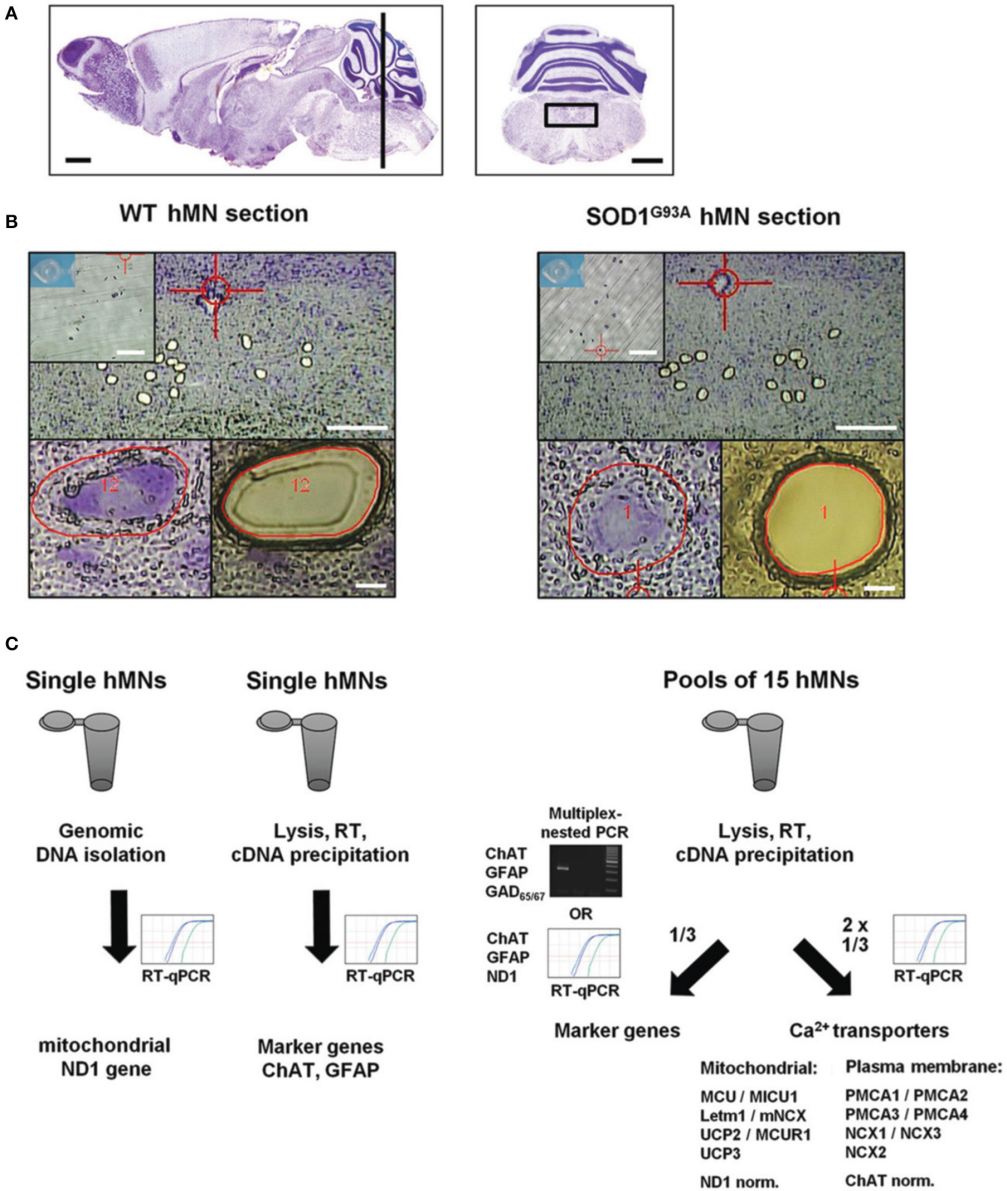
**Scheme of UV-LMD and RT-qPCR protocol for quantitative expression analysis of Ca^2+^ transporters in individual hypoglossal motor neurons from endstage SOD1^G93A^ and WT mice. (A)** Nissl-stained sagittal (left) adult mouse brain section. The black bar indicates the zone (6–7 mm posterior to the Bregma) within the brainstem, where coronal sections (right) containing hypoglossal motor neurons (hMNs) (black box) were cut for UV-LMD. Scale bars: 1 mm. Pictures taken from Paxinos and Franklin (2001). **(B)** Upper: Overview of a WT (left) and endstage SOD1^G93A^ mouse (right) coronal brainstem section after UV-LMD of 15 individual hMNs each. Scale bars: 250 μm. Inserts: photograph of the reaction tube cap for inspection of proper collection of all 15 neurons after UV-LMD, prior to cell lysis and reverse transcription (RT). Scale bars: 500 μm. Lower: individual hMNs before and after UV-LMD. Scale bars: 10 μm. **(C)** Workflow after UV-LMD. Left: genomic ND1 DNA-copy number of single hMNs was determined via qPCR with using genomic DNA as template, after genomic DNA isolation. ChAT and GFAP cDNA levels were determined for single hMNs after cell-lysis via individual RT-qPCR reactions with 50% of single cell cDNA each as templates. Right: For quantification of Ca^2+^ transporter expression-levels, cDNA derived from pools of 15 hMNs each were splitted, and 1/3 was used for marker gene expression profiling (either multiplex-nested PCR for ChAT, GFAP, and GAD_65/67_, or alternatively individual qPCRs for ChAT, GFAP, and ND1), and 2/3 was used for RT-qPCRs for quantification of Ca^2+^ transporter mRNA levels. For details, please see methods.

Carried out essentially as described (Fuchs et al., 2013; Schlaudraff et al., 2014). Briefly, SOD1^G93A^ and WT mice were deeply anesthetized with isoflurane (Abbott, Wiesbaden, Germany) and decapitated. Coronal tissue blocks containing hypoglossal nuclei were separated. The blocks were mounted on a specimen disk and immediately frozen by insertion into the snap-freeze holder (−35°C) of a cryostat (Leica CM 1850). Twelve μm serial coronal brainstem sections (for exact location see Figure 1A) were cut using a microtome blade (type R35, Feather, Osaka, Japan), and mounted on 2 mm PEN-membrane slides (Microdissect, Herborn, Germany), fixed with an ascending ethanol series, stained with cresyl violet, dried and stored at −80°C. UV-LMD of individual hMNs was performed using a Leica LMD7000 setup. 10 pools of 15 hMNs each were laser microdissected from each endstage SOD1^G93A^ and age-matched WT mouse. After cell-lysis and reverse transcription (RT) with random hexamer primers, cDNA was ethanol precipitated as described (Liss, 2002), resolved in 17 μl molecular biology grade water and stored at −20°C until PCR amplification. Note that hMNs of SOD1^G93A^ mice were about 5% larger than hMNs of WT control mice, according to area-quantifications after UV-LMD.

### Qualitative and quantitative PCR

Qualitative and quantitative PCR was carried out, essentially as described (Gründemann et al., 2011; Dragicevic et al., 2014; Schlaudraff et al., 2014). Quantitative PCR (qPCR) was carried out using TaqMan assays and a GeneAmp 7900HTqPCR cycler (Applied Biosystems, Darmstadt, Germany). All qPCR assay details and assay-specific standard curve parameters are given in Table 1. Standard curves were generated using serial dilutions of cDNA (stock concentration: 750 ng/μl, RNA integrity number (RIN): 9.8; derived from brainstem mRNA of an age-matched C57Bl/6 mouse). Five μl of standard cDNA or of the purified hMN cDNA was used as a template for each qPCR in a final volume of 20 μl, using QuantiTect Probe PCR Master Mix (Qiagen, Hilden, Germany), 1 μl TaqMan assay, and the following cycling conditions: 60°C 2 min, 95°C 15 min, (94°C 15 s, 60°C 1 min) 50 cycles.

**Table 1:** **Mouse TaqMan quantitative PCR assay information**.

				**Standard curve data**					
**Assay ID**	**Target gene reporter-context sequence-quencher (primer sequences)**	**Genbank accession no. (NCBI)**	**Ampl. Length [bp]**	**Exon spanning**	**Threshold**	**Y-Intercept**	**Slope**	***R*^2^**	***n***
Mm01168774_m1	MCU (Ccdc109a) FAM-CACCAAAGAGAGACCTCCTAAGCCA-NFQ	NM_001033259.3	92	4–5	0.6	45.77 ± 0.13	−3.43 ± 0.02	0.99 ± 0.00	8
Mm01173692_m1	MICU1 (Cbara1) FAM-AGACAGAAAAGTGATGGAGTATGAG-NFQ	NM_144822.2	67	3–4	0.6	44.73 ± 0.16	−3.39 ± 0.05	0.99 ± 0.00	8
Mm01351581_m1	MCUR1 (Ccdc90a) FAM-AAAGCAACAAGTGATGGATGAAGTG-NFQ	NM_001081059.3	98	5–6	0.6	46.13 ± 0.18	−3.14 ± 0.00	0.99 ± 0.01	2
Mm00522265_m1	Letm1 FAM-GCCAGCTGAAACAGTGGCTGGACTT-NFQ	NM_019694.1	74	7–8	0.6	44.30 ± 0.44	−3.31 ± 0.11	1.00 ± 0.00	3
Mm01197102_m1	mNCX (Slc24a6)^*^ FAM-TAGTCAAGTTGCCTGTGGAGTTCTT-NFQ	NM_133221.2, NM_001177594.1, NM_001177595.1	61	9–10/10–11	0.6	49.70 ± 0.71	−3.68 ± 0.17	0.98 ± 0.01	3
Mm01274107_g1	UCP2 FAM-GGTCCGGCTGCAGATCCAAGGGGAG-NFQ	NM_011671.4	82	3–4	0.6	44.72 ± 0.18	−3.33 ± 0.06	0.99 ± 0.00	4
Mm00494077_m1	UCP3 FAM-GTCTCACCTGTTTACTGACAACTTC-NFQ	NM_009464.3	69	5–6	0.6	48.62 ± 0.48	−3.33 ± 0.11	0.98 ± 0.01	5
Mm01245805_m1	PMCA1 (Atp2b1) FAM-GGGGACCTTACTCTGGGGCCAGCTT-NFQ	NM_026482.2	77	19–20	0.6	44.85 ± 0.32	−3.33 ± 0.09	0.99 ± 0.01	3
Mm00437640_m1	PMCA2 (Atp2b2)^*^ FAM-ATAGGCAAGGCGGGCCTGGTGATGT-NFQ	NM_001036684.2, NM_009723.3	79	7–8	0.6	42.32 ± 0.19	−3.29 ± 0.04	0.99 ± 0.00	4
Mm00623641_m1	PMCA3 (Atp2b3) FAM-AGACAAGAAAGGCAAGCAGCAGGAT-NFQ	NM_177236.3	71	6–7	0.6	43.94 ± 0.31	−3.30 ± 0.08	1.00 ± 0.00	3
Mm01285597_m1	PMCA4 (Atp2b4)^*^ FAM-TGAAAACCTCCCCTATAGAAGGTCT-NFQ	NM_213616.3, NM_001167949.1	86	1–2	0.6	46.86 ± 0.25	−3.23 ± 0.05	0.99 ± 0.01	3
Mm01232255_m1	NCX1 (Slc8a1)^*^ FAM-ACTGTCAGCGCTGGGGAAGATGACG-NFQ	NM_001112798.1, NM_011406.2	65	7–8/9–10	0.6	45.60 ± 0.38	−3.38 ± 0.06	0.99 ± 0.00	3
Mm00455836_m1	NCX2 (Slc8a2) FAM-AGGTGTAGTCCAGGTGTGGGAGGCA-NFQ	NM_148946.2	100	2–3	0.6	46.75 ± 0.24	−3.26 ± 0.08	0.98 ± 0.02	3
Mm00475520_m1	NCX3 (Slc8a3)^*^ FAM-CATCACTGTTAGTGCAGGAGGGGAT-NFQ	NM_080440.3, NM_001167920.1	71	5-6/6–7	0.6	46.14 ± 0.53	−3.28 ± 0.13	0.99 ± 0.00	3
Mm01221882_m1	ChAT FAM-TAGCTGTGAGGAGGTGCTGGACTTA-NFQ	NM_009891.2	67	3–4	0.8	46.13 ± 0.64	−3.04 ± 0.10	0.99 ± 0.00	3
Mm01253033_m1	GFAP^*^ FAM-AGAAAACCGCATCACCATTCCTGTA-NFQ	NM_001131020.1, NM_010277.3	75	6–7	0.8	42.41 ± 0.10	−3.29 ± 0.04	0.99 ± 0.00	3
Mm04225274_s1	ND1 FAM-ACAACCATTTGCAGACGCCATAAAA-NFQ	NC_005089_ND1.0	81	–	1.0	35.60 ± 0.56	−3.34 ± 0.03	1.00 ± 0.00	3

Threshold, slope and y-intercept values were determined with using serial dilutions of cDNA derived from mouse brainstem tissue as qPCR templates (for details, please see methods).

(*) Primers did detect but not discriminate between described splice variants.

**Figure 2:**
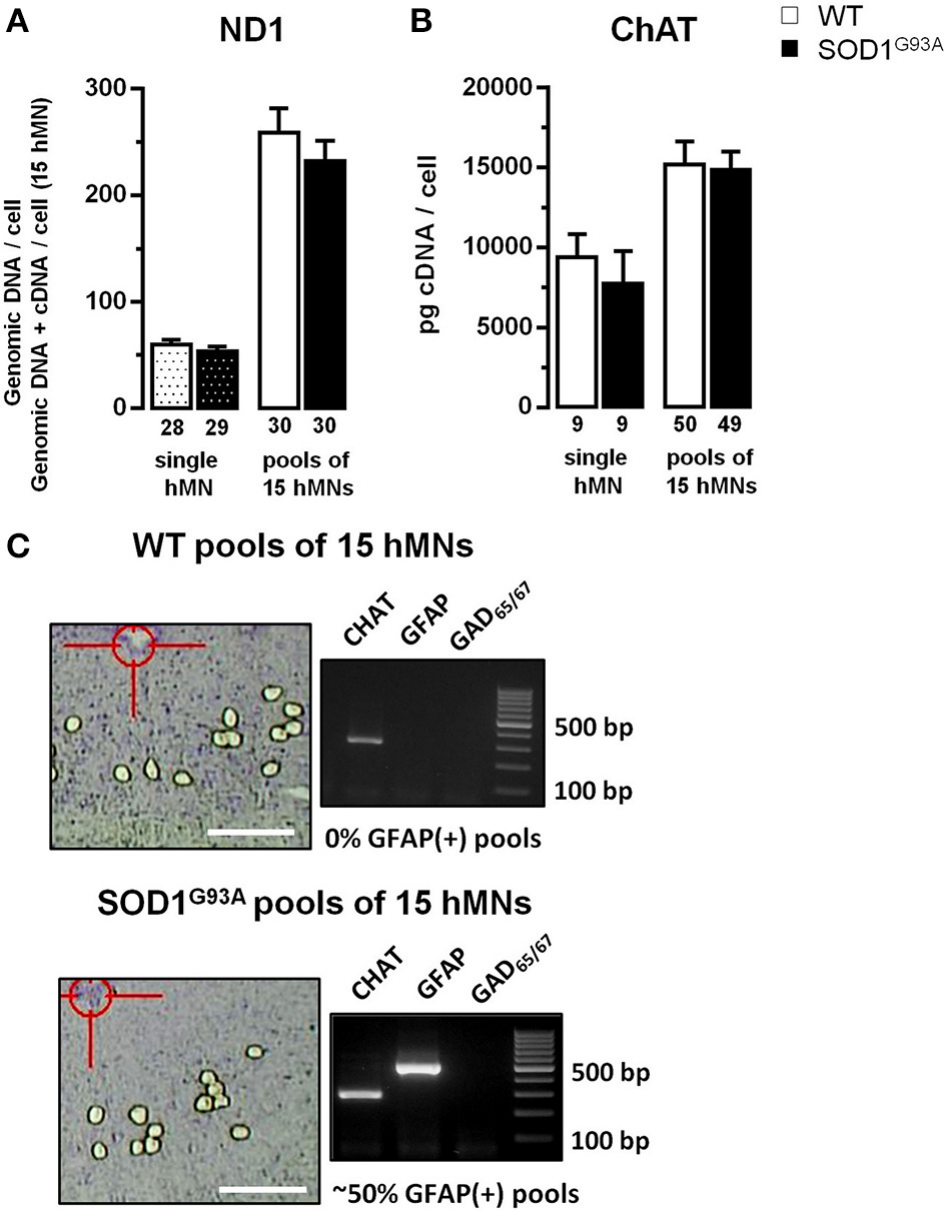
**Evaluation of ND1 and ChAT expression, used for normalization of cell-specific hypoglossal motor neurons qPCR data. (A)** qPCR data for mitochondrially coded NADH dehydrogenase ND1, using genomic DNA from individual hMNs as templates, indicate similar numbers of mitochondrial genomic DNA molecules in hMNs from WT and from endstage SOD1^G93A^ mice. Note that combined mitochondrial genomic DNA and cDNA levels for ND1, determined via RT-qPCR of pools of 15 hMNs each, are also not different between WT and SOD1^G93A^ mice, a prerequisite for using ND1 DNA-levels for normalization of respective mitochondrial Ca^2+^ transporter RT-qPCR results. **(B)** RT-qPCR data for choline-acetyltransferase (ChAT), derived either from single hMNs or from pools of 15 hMNs each, are similar in WT and SOD1^G93A^ mice, a prerequisite for using ChAT cDNA-levels for normalization of respective plasma membrane Ca^2+^ transporter RT-qPCR results. **(C)** WT and SOD1^G93A^ coronal brainstem section after UV-LMD of 15 individual hMNs and agarose gel products of multiplex-nested RT-PCR of the marker genes ChAT, GFAP (glial fibrillary acidic protein), and GAD_65/67_(L-glutamate decarboxylase). Note that most WT pools (or individual hMNs) were GFAP-negative while GFAP was robustly detected in ~50% of SOD1^G93A^ pools (or via qPCR in 100% of individual hMNs or pools from SOD1^G93A^, compare Table 2A). Scale bars: 250 μm.

To test for absence of contaminations in harvested hMN pools, 5 μl of purified hMN cDNA was subjected to either multiplex-nested PCR for qualitative (essentially as described, Dragicevic et al., 2014) or to qPCR for quantitative analysis of marker gene expression: We chose choline-acetyltransferase (ChAT) as marker for motor neurons, L-glutamate decarboxylase (GAD_65/67_) as marker for GABAergic cells, and glial fibrillary acidic protein (GFAP) as marker for astroglia cells. In addition, we used NADH dehydrogenase subunit 1 (ND1) as marker for mitochondrial genomic DNA copies. Only ChAT positive and GAD_65/67_ negative pools were further processed for Ca^2+^ transporter mRNA quantification. Please note that we detected in pools (~50–100%) as well as in individual hMN (100%) from SOD1^G93A^ but not from WT mice (0 and 11% respectively) consistently robust GFAP signals (compare Figure 2A and Table 2A). As GFAP positive and GFAP negative hMN pools from SOD1^G93A^ showed however no significant differences in MCU/MICU1 Ca^2+^ transporters expression levels, GFAP positive hMN pools were included into the analysis. Figure 1 illustrates the UV-LMD and RT-PCR workflow.

Table 2**Marker gene and Ca^2+^ transporter mRNA-levels in individual, laser microdissected hMNs from WT and endstage SOD1^G93A^ mice**.

**Table:** 

**A**							
	**Relative mRNA amount [pg/cell]**						
	**wildtype**			**SOD1^G93A^**			
	**Mean**	**±s.e.m.**	***n***	**Mean**	**±s.e.m.**	***n***	***p***
ChAT (15 hMNs)	15,196	1452	50	14,853	1156	49	0.80
ChAT (single hMN)	9411	1405	10	7743	2012	10	0.35
GFAP (15 hMNs)	**1.85**	**0.50**	**50**	**80.38**	**8.46**	**50**	<0.001^***^
GFAP (single hMNs)	**0.49**	**0.49**	**10**	**87.62**	**15.77**	**10**	<0.001^***^
ND1^†^ (15 hMNs)	258.5	23.22	30	232.0	18.82	30	0.51
ND1^#^ (single hMN)	60.32	3.64	28	53.91	3.73	29	0.23

**Table:** 

**B**														
	**(1) Relative mRNA amount [pg/cell]**							**(2) Relative amounts, normalized to ND1 levels [pg/cell]**						
	**Wildtype**			**SOD1^G93A^**				**wildtype**			**SOD1^G93A^**			
	**Mean**	**±s.e.m.**	***n***	**Mean**	**±s.e.m.**	***n***	***p***	**Mean**	**±s.e.m.**	***n***	**Mean**	**±s.e.m.**	***n***	***p***
MCU	13.17	2.98	8	23.88	4.00	10	0.08	**13.57**	**2.98**	**8**	**25.29**	**4.41**	**10**	0.04^*^
MICU1	**41.18**	**8.02**	**10**	**68.90**	**3.80**	**8**	0.03^*^	**38.79**	**6.28**	**10**	**69.95**	**8.80**	**8**	0.02^*^
MCUR1	4.90	1.03	9	5.24	0.90	10	0.70	4.69	0.70	9	6.13	0.83	10	0.39
LETM1	36.78	6.32	15	53.89	7.13	16	0.08	**31.86**	**3.64**	**9**	**54.25**	**7.26**	**10**	0.01^*^
UCP2	**3.27**	**0.61**	**15**	**5.48**	**0.73**	**14**	0.03^*^	**3.79**	**0.74**	**10**	**6.85**	**0.82**	**9**	0.01^*^
UCP3	–	–	5	–	–	5	–	–	–	5	–	–	5	–
MNCX	4.33	1.42	10	4.22	0.77	10	0.47	3.83	0.74	10	4.41	0.60	10	0.39
								**(3) Relative amounts, normalized to ChAT levels [pg/cell]**						
PMCA1	84.42	12.52	10	76.55	19.11	10	0.39	87.15	5.32	8	98.53	11.90	8	0.43
PMCA2	197.30	46.89	9	126.0	41.11	9	0.31	178.4	20.07	7	140.2	20.20	7	0.46
PMCA3	75.60	17.34	10	61.33	12.16	10	0.57	78.76	10.82	6	85.28	14.58	6	0.68
PMCA4	12.08	2.88	16	8.58	1.54	16	0.58	23.56	7.45	6	18.69	3.66	6	0.68
NCX1	19.39	3.05	10	20.24	3.89	10	0.95	**15.99**	**1.96**	**6**	**28.07**	**4.48**	**6**	0.03^*^
NCX2	–	–	5	–	–	5	–	–	–	5	–	–	5	–
NCX3	21.46	3.31	10	21.39	6.84	10	0.24	24.89	5.24	6	28.47	10.86	6	0.79

**(A)** RT-qPCR data for the marker-genes ChAT, GFAP, and ND1, derived from either pools of 15 hMNs, or from one individual hMN, given as pg/cell in respect to cDNA standard curves, generated from WT mouse brainstem tissue. Note that ND1 DNA-levels for individual hMNs

(#) were derived from isolated genomic DNA (without RT), while ND1 DNA-levels from pools of 15 hMNs

(†) were derived from cDNA + genomic DNA. Note that in contrast to WT, via qPCR, GFAP was robustly detected in about 100% of ChAT positive hMNs of endstage SOD1^G93A^ mice (compare Figure 2C). **(B)** RT-qPCR data for mitochondrial and plasma membrane Ca^2+^ transporters, derived from pools of 15 hMN each. Data are given (1) as [pg/cell] in respect to a cDNA standard curve, generated from WT mouse brainstem tissue, and (2) normalized to mitochondrially coded ND1 DNA-levels for mitochondrial Ca^2+^ transporters, or (3) normalized to ChAT cDNA levels for plasma membrane Ca^2+^ transporters. Significant differences according to Mann-Whitney-U-Tests are marked in bold and with

(*) , as defined in methods section.

### Determination of mitochondrial genome copy number

For quantification of mitochondrial genome copy number, single hMNs were laser microdissected and DNA isolation was performed with the QiaAmp DNA Micro Kit (Qiagen) according to the manufacturer protocol with the following adaptations: 0.1 μg/μl polyA carrier-RNA (included in kit) was added to each reaction, and all mixing steps were performed by pipetting with cell saver tips (Kisker, Steinfurt, Germany) to minimize sheering stress. DNA was eluted in 30 μl of molecular biology grade water and stored at 4°C until PCR amplification. For determination of mitochondrial DNA copy numbers, the mitochondrially coded ND1 gene was quantified, that is almost never affected by genomic deletion (He et al., 2002; Bender et al., 2006; Krishnan et al., 2007). Five μl of the eluted genomic DNA was used in a 20 μl reaction with QuantiTect Probe PCR Master Mix (Applied Biosystems), 1 μl ND1 TaqMan Primer Probe Mix, and 4 μl H_2_O for qPCR amplification in duplicate reactions in a GeneAmp 7900HT using the following cycling conditions: 60°C 2 min, 95°C 15 min, (94°C 15 s, 60°C 1 min) 50 cycles. ND1 qPCR assay details, and assay-specific standard curve parameters are given in Table 1.

### Data analysis

Data analysis, graphical representations, correlation and linear regression analysis were performed with SDS2.3 software (Applied Biosystems) and GraphPad Prism 6 (GraphPad Software Inc., San Diego, US). The cDNA amount per neuron in relation to the utilized standard was calculated as described (Gründemann et al., 2011; Schlaudraff et al., 2014) according to:

$$cDNA\;amount\;per\;cell=\frac{S^{\left[(Ct-Y_{intercept})slope\right]}}{No_{cells}•cDNA\;fraction}$$

With *S* = serial dilution factor of the standard curve (i.e., 10), *No*_*cells*_ = number of harvested neurons per UV-LMD sample (i.e., 15), *cDNA fraction* = fraction of the UV-LMD cDNA-reaction sample used as template in the individual qPCR reactions (pools of 15 hMNs: 5/17 for Ca^2+^ transporters, and 1/9 for marker genes ChAT, GFAP, and ND1; single hMN: 1/2 for marker genes ChAT and GFAP, and 1/6 for genomic ND1). The *Y*_*intercept*_ unit-magnitude corresponds to the respective standard utilized (i.e., pg equivalents of standard cDNA, derived from brainstem tissue mRNA). Single cell cDNA amounts were calculated with a *Y*_*intercept*_ of 42 for all Ca^2+^ transporter genes, and with *Y*_*intercept*_ from respective standard curves for ChAT, GFAP and ND1. Relative expression data are given as mean ± s.e.m., without and with normalization to ND1 and ChAT DNA/cDNA levels, respectively. Normalization was carried out by dividing respective Ca^2+^ transporter expression values to respective relative ChAT or ND1 expression values (relative to the WT mean values for ChAT and ND1), for each individual hMN pool. For statistical comparison Mann-Whitney-U-Tests were used. Significant differences are indicated by asterisks (^*^p < 0.05, ^**^p < 0.01, and ^***^p < 0.001).

## Results

To analyze expression levels of mitochondrial as well as plasma membrane Ca^2+^ transporters in individual hMNs from WT and endstage SOD1^G93A^ mice with best possible stratification, we further optimized our established single cell UV-LMD RT-qPCR protocol (Gründemann et al., 2011; Fuchs et al., 2013; Schlaudraff et al., 2014). Figure 1 summarizes the general work flow.

For cell-specific normalization and stratification of mitochondrial Ca^2+^ transporter mRNA expression-levels, we utilized the NADH dehydrogenase ND1 gene, that is encoded by mitochondrial genomic DNA, and is almost never affected by genomic DNA-degradation (He et al., 2002; Bender et al., 2006). Quantifying ND1 DNA copies in parallel with mitochondrial Ca^2+^ transporter mRNAs in individual hMNs allows the normalization of mitochondrial Ca^2+^ transporter expression levels to the number of mitochondria/mitochondrial genomes in the respective analyzed hMN pools. To probe if the number of mitochondrial genomes is altered in individual hMNs from WT and SOD1^G93A^ mice, we first quantified ND1 genomic DNA copy numbers after isolation of genomic DNA from individual hMNs. We detected no significant difference in ND1 genomic DNA levels in individual hMNs from WT and SOD1^G93A^ mice (Figure 2A and Table 2A). To probe if this is also the case, when analyzing (intronless) genomic ND1 levels without a distinct genomic DNA isolation step by using our established UV-LMD RT-qPCR protocol (a prerequisite for using this gene and this approach for RT-qPCR data normalization), we quantified ND1 DNA levels (genomic DNA + cDNA) in respective pools of 15 hMNs from WT and SOD1^G93A^ mice. Again, we detected no difference between hMNs from WT and SOD1^G93A^ mice, but as expected, about 4-fold higher ND1 DNA levels (genomic DNA + cDNA) per cell (WT: 4.28; SOD1^G93A^: 4.30) (Figure 2A and Table 2A).

For normalization and stratification of plasma membrane Ca^2+^ transporter expression levels, we utilized the cytoplasmic key enzyme for acetylcholine synthesis, the choline-acetyltransferase (ChAT). Again, as a prerequisite for using ChAT for cell-specific normalization of hMN expression data, we determined ChAT mRNA-levels of individual hMNs as well as of pools of hMNs in WT and endstage SOD1^G93A^ mice. Similar, as for ND1, we detected no difference in ChAT mRNA-levels in single or pooled individual hMNs from WT and SOD1^G93A^ mice (Figure 2B and Table 2A). However, as expected, we detected a strong correlation of ChAT mRNA-levels with individual hMN cell sizes (WT: *R*^2^ = 0.45; *p* < 0.001; *n* = 50; SOD1^G93A^: *R*^2^ = 0.48; *p* < 0.001; *n* = 49), further indicating the suitability of ChAT for normalization of plasma membrane Ca^2+^ transporter expression data from individual hMNs.

To ensure homogeneity of laser microdissected hMN pools, we analyzed for all hMN cDNA pools from WT and endstage SOD1^G93A^ mice (*n* = 32 and 55, respectively) a respective marker-gene expression profile. We utilized either qualitative RT-multiplex nested PCR for ChAT, the astroglia-marker GFAP (glial fibrilaric acidic protein) and the GABAergic neuron markers GAD_65_ and GAD_67_(L-glutamate-decarboxylase) (Figures 1C, 2C), or qPCR for ChAT and GFAP (Figure 1C and Table 2A). Only ChAT positive and GAD_65/67_ negative hMN pools were further analyzed. All ChAT positive hMN pools from WT or SOD1^G93A^ mice were negative for GAD_65/67_. However, as illustrated in Figure 2C, while WT pools showed no signal for GFAP, we obtained positive GFAP RT-PCR signals in about 50% of multiplex-nested PCRs of hMN cDNA pools from SOD1^G93A^ mice. To further address this unexpected finding, we quantified GFAP expression levels in pools of 15 hMNs as well as in individual hMN of WT and SOD1^G93A^ mice after UV-LMD. Again, we almost never detected any robust signal for GFAP in individual WT hMNs (*n* = 1 of 9 neurons), but in all 9 tested individual hMNs from SOD1^G93A^ mice, and in 100% (*n* = 50) of hMN pools (GFAP qPCR expression levels are given in Table 2A). Given these results, it is very unlikely that GFAP positive hMNs, selectively in SOD1^G93A^ mice are caused artificially due to technical issues. We next compared MCU and MICU1 mRNA-levels between GFAP positive and GFAP negative hMN pools of SOD1^G93A^ mice, and detected no significant difference (SOD1^G93A^, relative expression; MCU: GFAP pos. 1.71 ± 0.19, *n* = 16; GFAP neg. 1.65 ± 0.16, *n* = 23; *p* = 0.96; MICU1: GFAP pos. 1.98 ± 0.22, *n* = 16; GFAP neg. 1.78 ± 0.15, *n* = 23; *p* = 0.51). Thus, we did not exclude GFAP positive hMN pools from further PCR analysis of Ca^2+^ transporter expression.

**Figure 3:**
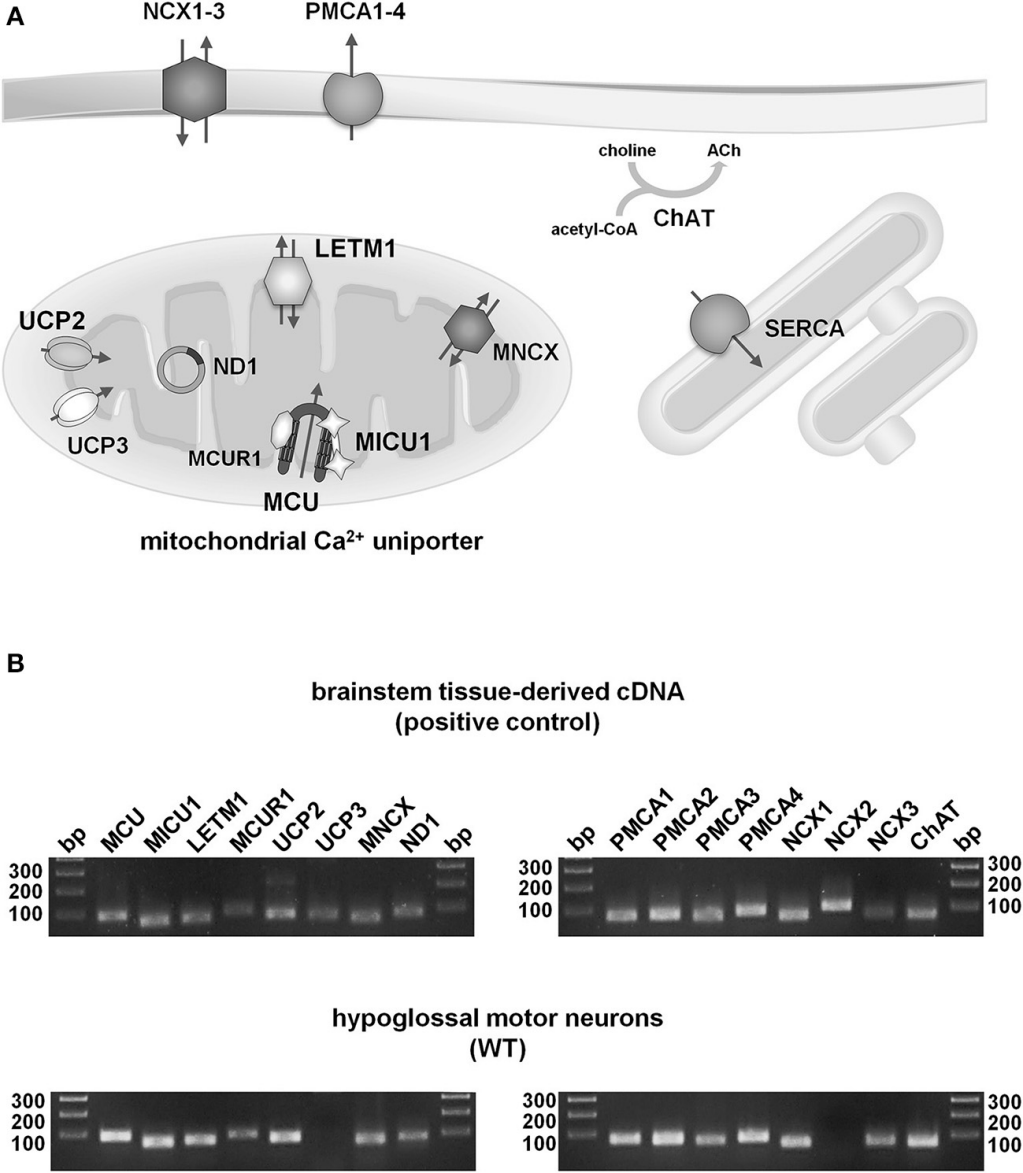
**Qualitative expression profiling of putative mitochondrial and plasma membrane Ca^2+^ transporters in individual hypoglossal motor neurons from WT and endstage SOD1^G93A^ mice. (A)** Illustration of proteins for main putative mitochondrial (MCU/MICU1/MICUR1, Letm1, UCP2/3, MNCX), plasma membrane (PMCA1-4, NCX1-3) and ER (SERCA) Ca^2+^ transporters in cholinergic (ChAT) hMNs. For details and abbreviations, please see text. **(B)** Upper: Agarose gel (2%) electrophoresis of RT-qPCR products indicates that all putative mitochondrial and plasma membrane Ca^2+^ transporters, as well as ChAT and ND1, are expressed in cDNA, derived from mouse brainstem tissue (positive control). Lower: Agarose gel electrophoresis of RT-qPCR products shows that all putative mitochondrial and plasma membrane Ca^2+^ transporters, but UCP3 and NCX2, are expressed in ChAT and ND1 positive hMNs from WT mice (as well as from SOD1^G93A^ mice, not shown) (bp: base pairs, DNA ladder).

**Figure 4:**
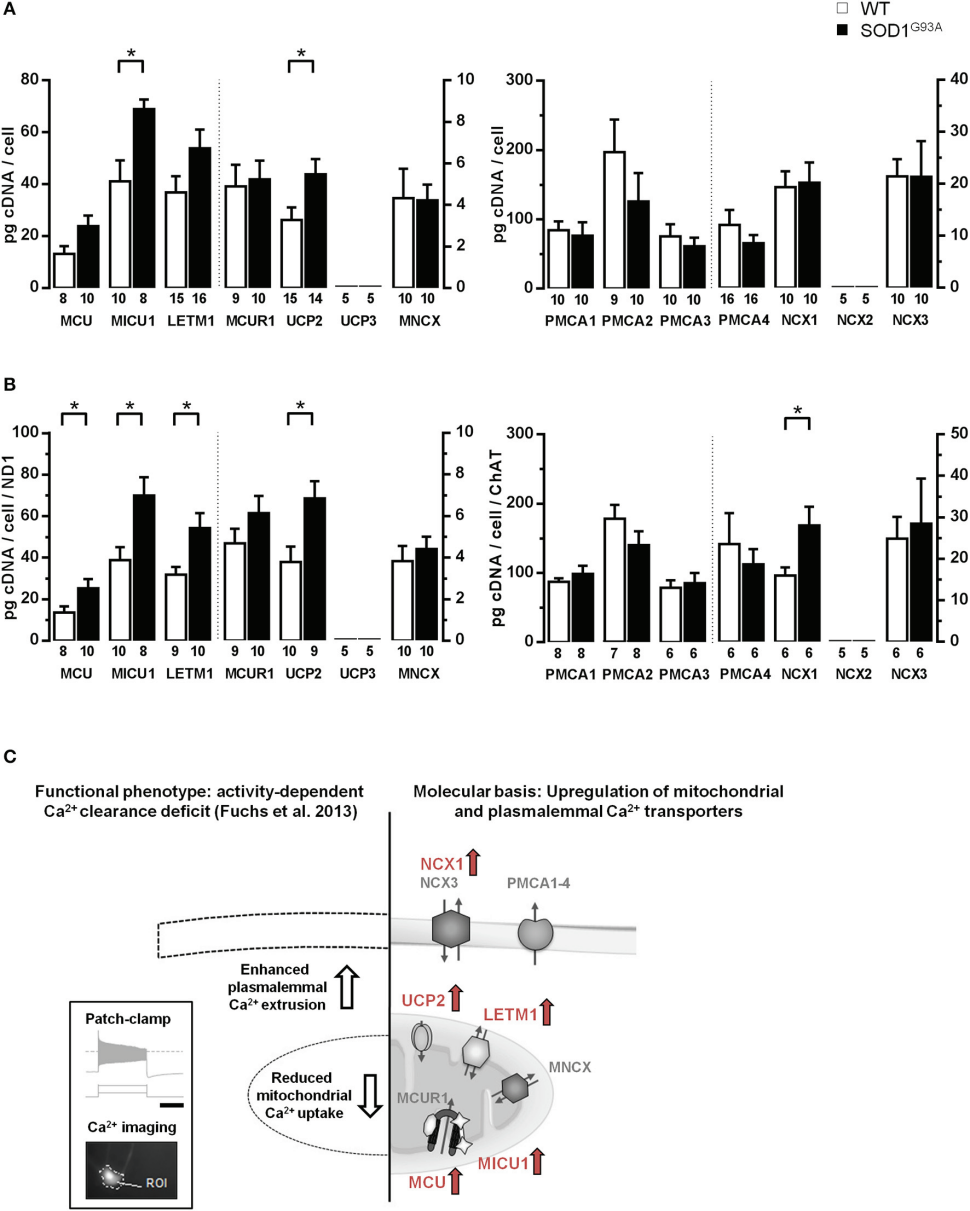
**Elevated mRNA-levels of distinct Ca^2+^ transporters in individual hypoglossal motor neurons from SOD1^G93A^ mice compared to WT. (A)** Cell-specific RT-qPCR data for mitochondrial and plasma membrane Ca^2+^ transporters, derived from pools of 15 hMNs each from SOD1^G93A^ mice and WT. Data are given as [pg/cell] in respect to a cDNA standard curve, generated from WT mouse brainstem tissue. **(B)** Data from **(A)**, normalized to mitochondrially coded ND1 DNA-levels for mitochondrial Ca^2+^ transporters, and to ChAT cDNA levels for plasma membrane Ca^2+^ transporters. Significant differences according to Mann-Whitney-U-Tests are marked with (^*^), as defined in methods section. Note that data in **(A,B)** for the respective genes refer to two different axes, as indicated by the dashed line. **(C)** Right: Overview of concerted elevated Ca^2+^ transporter expression in individual hMN from SOD1^G93A^ mice compared to WT (marked in red: MCU/MICU1, Letm1, UCP2, and NCX1). Left: These findings can provide a molecular basis for the recently described, selective functional, activity-dependent Ca^2+^ homeostasis deficit in hMN from endstage SOD1^G93A^ mice. Insert shows stimulation protocol and resulting electrophysiological recordings (scale bar: 1 s), combined with calcium imaging (for details please see text and Fuchs et al., 2013).

Next, via RT-qPCR, we analyzed qualitative mRNA expression of the main described potential mitochondrial and plasma membrane Ca^2+^ transporters (Figure 3A) in pools of 15 hMNs from WT and SOD1^G93A^ mice. As illustrated in Figure 3B, mRNAs for all tested mitochondrial Ca^2+^ transporters (MCU/MICU1/MCUR1, Letm1, UCP2, MNCX) but UCP3, and for all plasma membrane Ca^2+^ transporters (PMCA1-4, NCX1, NCX3) but NCX2, were detected in ChAT and ND1 positive hMNs (*n* = 3 pools of 15 neurons each) from WT and SOD1^G93A^ mice. Accordingly, we quantified mRNA-levels for all these mitochondrial and plasma membrane Ca^2+^ transporters via qPCR in hMNs from WT and endstage SOD1^G93A^ mice. Results are given in Figure 4 and Table 2B. Without cell-specific normalization, we detected significantly higher mRNA levels of only MICU1 and UCP2 in hMNs from SOD1^G93A^ mice (each about 1.7-fold higher compared to WT; Figure 4A left and Table 2B). All other Ca^2+^ transporter mRNA-levels were not significantly altered (although trends were observed in particular for MCU and Letm1, compare Figure 4A and Table 2B). Accordingly, cell-specific normalization of hMN qPCR data for mitochondrial Ca^2+^ transporters to mitochondrially coded ND1 DNA-levels (and thus to respective hMN mitochondrial genome numbers) revealed significantly higher mRNA levels of not only MICU1 and UCP2, but also of MCU and Letm1 in hMNs from SOD1^G93A^ mice (each about 1.8-fold higher compared to WT), while levels of MCUR1 and MNCX were not altered (Figure 4B left and Table 2B). In addition, by cell-specific normalization of hMN qPCR data for all analyzed plasma membrane Ca^2+^ transporters to relative ChAT levels (and thus to respective hMN cell-sizes), we detected about 1.7-fold higher mRNA-levels, selectively of NCX1 in hMNs from SOD1^G93A^ mice compared to WT (Figure 4B right and Table 2B). All other tested plasma membrane Ca^2+^ transporters (PMCA1-4 and NCX3) were not altered in hMNs from SOD1^G93A^ mice compared to those of WT.

In summary (Figure 4C), we detected at the mRNA-level a concerted up-regulation of three distinct putative mitochondrial Ca^2+^ transporters, MCU/MICU1, Letm1 and UCP2, in individual hMNs from endstage SOD1^G93A^ mice compared to WT, likely causing altered Ca^2+^ uptake of diseased mitochondria in hMN from SOD1^G93A^ mice. The elevated expression of the plasma membrane Ca^2+^ transporter NCX1 provides a molecular explanation for the described functionally enhanced activity dependent intracellular Ca^2+^ extrusion via the plasma membrane in hMNs from SOD1^G93A^ mice (Fuchs et al., 2013).

## Discussion

Here we provide a cell-specific quantitative analysis of mitochondrial and plasma membrane Ca^2+^ transporter mRNA expression in highly vulnerable cholinergic hypoglossal motor neurons from SOD1^G93A^ transgenic mice compared to WT. We identified a selective up-regulation of the mitochondrial Ca^2+^ transporters MCU/MICU1, Letm1 and UCP2 as well as of the plasma membrane Na^+^/Ca^2+^ exchanger NCX1 in remaining hMNs in endstage SOD1^G93A^ mice, while cDNA and genomic DNA levels for the mitochondrially coded ND1 gene, as well as cDNA levels for ChAT, the key enzyme for acetylcholine-synthesis, were not altered.

SOD1^G93A^ transgenic mice are still the most widely used and most extensively characterized mouse-model for ALS (Vinsant et al., 2013a,b). These mice express a high copy number of the human G93A mutant SOD1, and recapitulate many key features of the human ALS phenotype, like adult disease onset, selective motor neuron degeneration and axonal loss (Gurney et al., 1994; Chiu et al., 1995; Fuchs et al., 2010). The limited clinical efficacy of compounds tested on SOD1 mice (Vucic et al., 2014), as well as the notion that SOD1 mutations account only for a small number (~2%) of sporadic ALS cases (Renton et al., 2013) prompted the generation of new rodent ALS-models, like mice with mutated or overexpressed TDP-43 or fused in sarcoma (FUS) (Da Cruz and Cleveland, 2011; Van Den Bosch, 2011; Wegorzewska and Baloh, 2011; McGoldrick et al., 2013). However, given our previous functional analysis of activity dependent Ca^2+^ homeostasis in hMNs from SOD1^G93A^ mice (Fuchs et al., 2013) and the fact that none of the recently generated models was shown to recapitulate most aspects of human ALS as convincing as the SOD1 mutant mouse, we continued to focus on the analysis of SOD1^G93A^ mice in this complementary study.

Although mitochondrial dysfunction is present in MNs in human ALS as well as in SOD1^G93A^ mice (Barrett et al., 2011; Martin, 2011), and macroscopic and functional alterations of mitochondria have been characterized extensively (Kawamata and Manfredi, 2010; Cozzolino and Carrì, 2012; Vehviläinen et al., 2014), the number of mitochondria/mitochondrial genomes in highly vulnerable MNs to our knowledge has not yet been addressed. Our cell-specific ND1 data (Figure 2A) argue against significant changes in number of mitochondria or mitochondrial genomes (at least in the cell soma) in hMNs from endstage SOD1^G93A^ mice, compared to WT. In accordance with our findings, ND2 genomic DNA levels in human *postmortem* spinal cord motor neurons of ALS patients were also not altered compared to controls, whereas mitochondrial genomic DNA-levels for mitochondrially coded cytochrome oxidase and ND4 were significantly lower in ALS (Keeney and Bennett, 2010)—probably due to mitochondrial DNA deletions that occur preferentially in the ND4 gene (He et al., 2002). These deletions or mutations are likely to occur more frequently in ALS, as DNA repair enzymes in mitochondria have been shown to be impaired (Murakami et al., 2007).

In addition, our cell-specific data identified mitochondrially coded ND1 as a well-suited gene for normalization and further stratification of RT-qPCR data from hMNs. This is particularly useful when analyzing cell-specific expression-levels of targets with gene-products located in mitochondria (like MCU/MICU1/MICUR1, Letm1, UCP2, and MNCX). Furthermore, we identified ChAT as a well-suited gene for normalization of RT-qPCR data for target-genes expressed in the cytoplasm or in the plasma membranes of hMNs (like PMCA1-4 and NCX1/3). Our data show, that this cell- and transporter-class-specific normalization approach allows stratification of single cell hMN expression data, and thus reduces the number of neurons or neuronal pools that need to be analyzed for detection of significant differences (compare Figures 4A,B, Table 2B and Fuchs et al., 2013); MCU here: *n* = 8 and 8; in Fuchs et al. without normalization *n* = 21 and 39 for WT and SOD1^G93A^ hMN pools, respectively).

### GFAP co-expression in ChAT-positive hMNs selectively from SOD1^G93A^ but not from WT mice?

Surprisingly, with multiplex-nested PCR, as well as with more sensitive qPCR, we detected GFAP co-expression in about 50 or 100% respectively, of analyzed ChAT positive hMN cDNA pools from SOD1^G93A^ mice but not in WT (Figure 2B and Table 2A). How to interpret these findings? As we can exclude general technical issues (i.e., contaminations), we provide two explanations for the detected GFAP signals selectively in hMNs from SOD1^G93A^ but not WT mice. Either SOD1^G93A^ hMNs might de-differentiate or reprogram their neuronal phenotype due to the disease process, as described for other cell types (Sarthy et al., 1991; Hol et al., 2003; Arendt, 2008; Puri and Hebrok, 2012; Qiang et al., 2013; Gao et al., 2014), and due to this process co-express GFAP. Or alternatively, the well-described reactive atsroglia cell activation and proliferation, present in ALS and other neurodegenerative diseases (Guan et al., 2007; Chen et al., 2012; Parpura et al., 2012; Forsberg et al., 2011; Bi et al., 2013), might change the morphological astroglia—neuron interaction, and thus, dendrites of astroglial cells might reside more closely to hMNs from SOD1^G93A^ compared to WT, and might have been partly laser microdissected together with the hMN cell bodies. However, it is important to note that we gained no morphological evidence for this latter GFAP contamination explanation in our UV-LMD samples from SOD1^G93A^ brains. Furthermore, we never detected respective GFAP co-expression in individual dopamine neurons from, e.g., Parkinson's disease brains, where a respective reactive gliosis also has been described ('Episcopo et al., 2013), or from respective Parkinson's disease mouse models, in over 15 years of UV-LMD RT-qPCR analysis of individual dopamine neurons (Ramirez et al., 2006; Gründemann et al., 2008, 2011; Schiemann et al., 2012; Schlaudraff et al., 2014). Thus, while we can rule out general methodological issues, we cannot for sure conclude that the detected GFAP signal of hMNs selectively from SOD1^G93A^ but not from WT mice is genuinely derived from hMNs, and further immunohistological studies are necessary to address this point and its possible implication for ALS. However, this surprising finding further highlights the emerging crucial role of astroglia cells in ALS and its animal models (Valori et al., 2014). Indeed mutant SOD1 expression has been shown to greatly affect the astroglial functional phenotype, turning astrocytes into neurotoxic cells, more prone to cell death, and altering their vital MN-supportive functions (Valori et al., 2014).

### Elevated mRNA levels of the mitochondrial Ca^2+^ transporters MCU/MICU1, Letm1 and UCP2 and the plasma membrane Na^+^/Ca^2+^ exchanger NCX1 in hMNs of SOD1^G93A^ mice

The present study was motivated by our recent finding of an activity-dependent Ca^2+^ clearance deficit selectively in individual hMNs, at the endstage of disease in SOD1^G93A^ mice (Fuchs et al., 2013). More precisely, by combining patch-clamp analysis with fura-2 calcium imaging and selective pharmacology (e.g., the MCU-inhibitor RU-360) of individual, highly vulnerable hypoglossal and mostly resistant oculomotor MNs, we identified a remodeling of activity-dependent, intracellular Ca^2+^ clearance, selectively in hMNs in SOD1^G93A^ mice at disease endstage, that was characterized by a reduction of mCU-mediated mitochondrial Ca^2+^ uptake, and an enhanced Ca^2+^ extrusion across the plasma membrane, under high-Ca^2+^ loading conditions (Fuchs et al., 2013). Our preliminary molecular analysis pointed to a complex underlying mechanism, as mRNA-levels of the mCU core components MCU and MICU1 were about 1.7-fold higher in hMNs from SOD1^G93A^ mice (Fuchs et al., 2013). With an improved UV-LMD RT-qPCR protocol (Figures 1, 2), we could reproduce these findings in an independent cohort of mice (Figures 4A,B; Table 1B). Increased MCU/MICU1 expression might enforce metabolic coupling, as mitochondrial Ca^2+^ activates, e.g., enzymes of the tricarboxylic acid cycle (McCormack and Denton, 1990; Wiederkehr et al., 2011). On the other hand, MICU1 does also act as gatekeeper of mCU that sets a threshold for maximal Ca^2+^ uptake. Thus, a MICU1 up-regulation provides a protective mechanism against mitochondrial calcium overload due to increased cytosolic Ca^2+^ levels (Mallilankaraman et al., 2012b), preventing excessive ROS generation and apoptosis (de Stefani et al., 2011). This mCU repressor-function of elevated MICU1 could explain the described functionally reduced mitochondrial Ca^2+^ uptake, while MICU1 and MCU mRNA levels are elevated. The finding that mRNA-levels of another regulatory mCU subunit MCUR1 (Mallilankaraman et al., 2012a) were not altered in hMNs from SOD1^G93A^ mice, further supports the idea that the stoichiometry and thus general function of mCU might indeed be altered in hMNs from SOD1^G93A^ mice. Additional mCU components have recently been identified (Marchi and Pinton, 2014), like EMRE and MICU2, which regulate MCU/MICU1 activity (Ahuja and Muallem, 2014; Kamer and Mootha, 2014; Kevin Foskett and Madesh, 2014), that need to be analyzed in further studies.

In addition to elevated MCU/MICU1 levels, we detected similarly elevated levels for Letm1 as well as for UCP2 mRNA in hMNs from SOD1^G93A^ mice, while UCP3 mRNA was neither detected in hMNs from WT nor from transgenes (Figures 4A,B; Table 1B). Letm1 is regarded as a highly Ca^2+^ sensitive mitochondrial Ca^2+^/H^+^ exchanger, operating mainly at cytosolic Ca^2+^ levels below 1 μM (Jiang et al., 2009; Waldeck-Weiermair et al., 2011). As significant mitochondrial Ca^2+^ uptake via the mCU becomes apparent particularly at cytosolic Ca^2+^ concentrations not below 5–10 μm (Rizzuto and Pozzan, 2006; Marchi and Pinton, 2014), a concerted up-regulation of mCU and Letm1 migth counteract pathophysiological activity dependent, altered cytosolic Ca^2+^ levels in SOD1^G93A^ mice, and thereby promote hMN survival. This complex view is further supported by the detected elevated levels of UCP2 in hMNs from SOD1^G93A^ mice. In addition to the proposed mediated direct mitochondrial Ca^2+^ uptake, UCP2 acts as mitochondrial uncoupling protein, and thus decreases the amount of reactive oxygen species produced by a steep proton gradient of the respiratory chain (Donadelli et al., 2014). This mild uncoupling has been shown to be neuroprotective in highly vulnerable dopamine neurons in mouse models of Parkinson's disease (Liss et al., 2005; Guzman et al., 2010). However, overexpression of human UCP2 in SOD1^G93A^ mice paradoxically accelerated disease progression and further reduced mitochondrial Ca^2+^ uptake capacity (Peixoto et al., 2013). The authors conclude, that in bio-energetically defective mitochondria of SOD1^G93A^ mice, UCP2 might have adverse effects, possibly by enhancing sensitivity to Ca^2+^ induced depolarization of mitochondria and/or interacting with other Ca^2+^ uptake pathways.

Mitochondrial Ca^2+^ uptake and Ca^2+^ transporter activity is closely related to ER Ca^2+^ uptake, predominantly mediated by SERCA (Hovnanian, 2007; Chaudhari et al., 2014; Hajnóczky et al., 2014). We did however not analyze mRNA expression levels of SERCA-isoforms, as our previous functional and pharmacological (thapsigargin) analysis gave no hint for altered ER Ca^2+^ uptake in hMNs of endstage SOD1^G93A^ mice, but for enhanced plasma membrane Ca^2+^extrusion (compare Figure 4C and Fuchs et al., 2013). In accordance with this functional phenotype, we detected an about 1.7-fold higher mRNA-level of the plasma membrane Na^+^/Ca^2+^ exchanger NCX1 in hMNs from SOD1^G93A^ mice, while NCX2 was not expressed, and NCX3 as well as PMCX1-4 mRNA-levels were not altered (Figure 4 and Table 2B). The lack of NCX2 mRNA detection in hMNs from WT as well as from SOD1^G93A^ mice is somewhat surprising, since this isoform was attributed a key role in neuronal Ca^2+^ homeostasis (Brini and Carafoli, 2011). The low-affinity/high-capacity Ca^2+^ transporter NCX is believed to mediate the bulk of plasma membrane Ca^2+^ export after stimulation, rather than the PMCAs (Brini and Carafoli, 2011; Sharma and O'Halloran, 2014). Moreover, NCXs are controlled by cytosolic concentrations of Na^+^ and Ca^2+^ (Brini and Carafoli, 2011), thus the observed increased functional plasma membrane Ca^2+^-extrusion is likely caused by an enhanced NCX1 expression, accompanied by an enhanced functional NCX transport capacity, due to pathologically elevated cytosolic Ca^2+^ and a reduced mitochondrial Ca^2+^ uptake capacity in hMNs from endstage SOD1^G93A^ mice. Vice versa, overexpression of NCX1 has been shown to reduce not only cytosolic but also mitochondrial Ca^2+^ transients, providing an additional feedback loop to protect mitochondria from an extensive Ca^2+^ overload (Brini et al., 2002). An up-regulation of NCX1 in neurons has been demonstrated to be neuroprotective in other pathologic conditions, e.g., ischemic events (Pignataro et al., 2012). However, PMCAs are also stimulated by Ca^2+^ via calmodulin or phosphorylation (Strehler, 2013; Lopreiato et al., 2014). As specific PMCA splice variants differ, e.g., in their affinity and reaction upon calmodulin binding (Brini et al., 2013; Strehler, 2013), an pathologic shift in PMCA splice variants might also contribute to enhanced plasma membrane Ca^2+^ extrusion in hMNs from SOD1^G93A^ mice. It is noteworthy in this context, that our PMCA TaqMan assays are detecting but not discriminating distinct PMCA splice variants. To date, functional studies of plasma membrane Ca^2+^ transporters are hampered by lack of specific pharmacology which makes it difficult to address the distinct contributions of individual NCX- or PMCA-isoforms (Strehler, 2013).

In conclusion, this study provides novel molecular and cell-specific insights into the complex nature of cytosolic Ca^2+^ dysregulation and mitochondrial dysfunction in highly vulnerable hMNs in the most common ALS mouse model, the endstage SOD1^G93A^ mice. The detected concerted up-regulation of distinct mitochondrial and plasma membrane Ca^2+^ transporters with different locations as well as transport kinetics (mCU, Letm1, UCP2, NCX1) might serve as a complex compensatory response to the disease trigger and to the altered Ca^2+^ homeostasis, and protect hMNs in SOD1^G93A^ mice from mitochondrial Ca^2+^ overload and degeneration. However, as we analyzed endstage SOD1^G93A^ mice, where already about 30% of hMNs are lost (Haenggeli and Kato, 2002), we do not know, if the molecular up-regulation of distinct Ca^2+^ transporters did indeed protect those remaining hMNs from degeneration.

## Author contributions

TM and JD carried out all experiments and performed data analysis, TM, JD and BL designed the study and wrote the manuscript. JHW and ACL bred and provided SOD1^G93A^ and WT mice and revised the manuscript.

### Conflict of interest statement

The authors declare that the research was conducted in the absence of any commercial or financial relationships that could be construed as a potential conflict of interest.
